# Diosgenin Ameliorated Type II Diabetes-Associated Nonalcoholic Fatty Liver Disease through Inhibiting De Novo Lipogenesis and Improving Fatty Acid Oxidation and Mitochondrial Function in Rats

**DOI:** 10.3390/nu14234994

**Published:** 2022-11-24

**Authors:** Yujie Zhong, Zhiman Li, Ruyi Jin, Yanpeng Yao, Silan He, Min Lei, Xin Wang, Chao Shi, Li Gao, Xiaoli Peng

**Affiliations:** 1 College of Food Science and Engineering, Northwest A&F University, Xianyang 712100, China; 2 Prescription Laboratory of Xinjiang Traditional Uyghur Medicine, Xinjiang Institute of Traditional Uighur Medicine, Urmuqi 830011, China

**Keywords:** Diosgenin, non-alcoholic fatty liver disease, de novo lipogenesis, fatty acid β-oxidation, mitochondrial fission and apoptosis

## Abstract

Diosgenin (DIO) is a dietary and phytochemical steroidal saponin representing multiple activities. The present study investigated the protective effect of DIO on type II diabetes-associated nonalcoholic fatty liver disease (D-NAFLD). The rat model was established by high-fat diet and streptozotocin injection and then administered DIO for 8 weeks. The results showed that DIO reduced insulin resistance index, improved dyslipidemia, and relieved pancreatic damage. DIO decreased hepatic injury markers, including aspartate aminotransferase (AST) and alanine aminotransferase (ALT). H&E staining showed that DIO relieved hepatic lipid deposition. Mechanistically, DIO inhibited hepatic de novo lipogenesis (DNL) and increased fatty acid β-oxidation (FAO) through regulation of the AMPK-ACC/SREBP1 pathway. Endoplasmic reticulum (ER) stress was inhibited by DIO through regulation of PERK and IRE1 arms, which may then inhibit DNL. DIO also decreased reactive oxygen species (ROS) and enhanced the antioxidant capacity via an increase in Superoxide dismutase (SOD), Catalase (CAT), and Glutathione peroxidase (GPx) activities. The mitochondria are the site for FAO, and ROS can damage mitochondrial function. DIO relieved mitochondrial fission and fusion disorder by inhibiting DRP1 and increasing MFN1/MFN2 expressions. Mitochondrial apoptosis was then inhibited by DIO. In conclusion, the present study suggests that DIO protects against D-NAFLD by inhibiting DNL and improving FAO and mitochondrial function.

## 1. Introduction

Type 2 diabetes mellitus (T_2_DM) is a kind of metabolic disease characterized by insulin resistance and persistent hyperglycemia [1]. Non-alcoholic fatty liver disease (NAFLD) and T_2_DM often co-exist and have mutual influence [2,3] It is reported that 50–75% of T_2_DM are diagnosed as NAFLD, which is two to three times as much as in the general population (25%) [4,5,6].

NAFLD is a common chronic liver disorder characterized by hepatic lipid deposition (>5%), which includes simple fatty liver with or without mild inflammation and steatohepatitis with necroinflammation and faster fibrosis progression [7,8]. NAFLD is a consequence of lipid acquisition exceeding lipid disposal. In this event, de novo lipogenesis (DNL) and the uptake of fatty acids exceed the fatty acid β-oxidation (FAO) and export [9]. Insulin resistance serves as one of the earliest pathogenic events in both NAFLD and T_2_DM, which results in hyperinsulinemia, increased free fatty acid (FFA), and DNL, followed by the accumulation of triglyceride (TG) [10,11]. Sterol regulatory element-binding protein 1 (SREBP1) is a key transcription factor for DNL and lipid accumulation, which upregulates genes coding for Acetyl-CoA carboxylase (ACC) and fatty acid synthase (FASN) [12]. FAO is the primary route of lipid depletion. AMP-activated protein kinase (AMPK) plays a crucial role in DNL and FAO. It can inhibit DNL and increase FAO through suppressing the cleavage of SREBP1 to mature SREBP1 and the conversion of phosphorylated ACC to inactive ACC [13].

Since the bulk of lipid synthesis takes place in the smooth endoplasmic reticulum (ER), ER stress involves in the development of hepatic steatosis. ER stress affects the process of NAFLD via regulating lip-stasis, modulating hepatic insulin sensitivity, and regulating hepatic autophagic flux. Growing evidence has suggested that chronic or acute ER stress will aggravate hepatic steatosis and hepatocyte death [14,15]. Thus, the regulation of ER stress is a therapeutic target for NAFLD. For example, Chen et al. [16] indicated that resveratrol supplementation prevented ER stress and mitigated hepatic steatosis and resultant damage in a murine model of ER stress.

The primary route of lipid depletion is FAO, which is carried by mitochondria. However, persistent FAO will produce massive ROS and then damage mitochondria due to the increase of FFA. Subsequently, the defective FAO appears [17]. At the same time, ROS damages mitochondrial DNA and weakens mitochondrial function. To maintain their shape and function, mitochondria undergo continuous fusion and fission [18]. Studies have indicated that enhanced mitochondrial fission or damaged mitochondrial fusion triggers hepatic steatosis, inflammation, and hepatocyte death through the caspase cascade [19,20]. The damage to mitochondrial DNA can also lead to the mitochondrial pathway of apoptosis. Therefore, improving mitochondrial dysfunction represents a novel therapeutic target for NAFLD.

Severe side effects have been reported to accompany long-term administration of drugs for lowering blood glucose and regulating lipid metabolism. Seeking natural active substances with low toxicity to prevent and treat NAFLD and T_2_DM has attracted increasing attention. Diosgenin (DIO) is a dietary and phytochemical steroidal saponin that displayed efficacy against various life-threatening diseases such as diabetes and its complications, hyperlipidemia, and cardiovascular diseases [21,22]. It is indicated that DIO ameliorated hepatic steatosis through inhibiting fatty acid synthesis [23]. However, whether the regulation of DNL, FAO, oxidative stress, and mitochondrial function contributes to the protective effect of DIO on type II diabetes-associated nonalcoholic fatty liver disease (D-NAFLD) is still unclear. Therefore, the purpose of the present study is to reveal whether DIO protects against D-NAFLD from the above points.

## 2. Materials and Methods

### 2.1. Animals and Experimental Design

**Figure 1:**
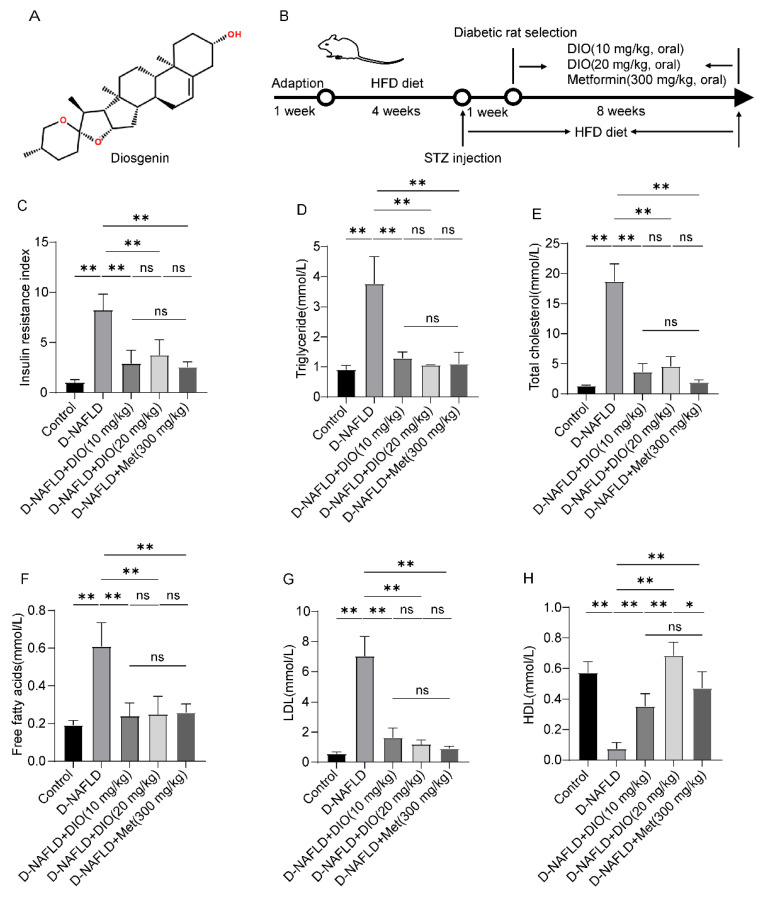
DIO reduced insulin resistance and improved dyslipidemia in D-NAFLD rats. (**A**) The structure of DIO. (**B**) Experimental design. (**C**) Insulin resistance index. (**D**) Serum triglyceride (mmol/L). (**E**) Serum total cholesterol (mmol/L). (**F**) Serum free fatty acids (mmol/L). (**G**) Serum LDL (mmol/L). (**H**) Serum HDL (mmol/L). *n* = 8 and the data were presented as mean ± SD. ns indicates no significance, * and ** indicate significant difference and highly significant difference, respectively.

Sprague Dawley male rats (200 ± 20 g) were obtained from Dossy Experimental Animals Co., Ltd. (Chengdu, Sichuan, China). After adaptation for 1 week, the rats were distributed into the control group (*n* = 8) and the T_2_DM group (*n* = 32). T_2_DM rats were established by high-fat diet (HFD, Dossy Experimental Animals Co., Ltd.) and streptozotocin (STZ, Solarbio Science & Technology Co., Ltd., Beijing, China) injection. Firstly, the rats were administrated with HFD for 4 weeks. The formula of HFD refers to our previous article [24]. Then they were given a single injection of STZ (35 mg/kg) [25]. Subsequently, the T_2_DM rats (random blood glucose > 300 mg/dL) were distributed into 4 groups (*n* = 8): D-NAFLD group, D-NAFLD+DIO (10 mg/kg, > 98% purity, Spring Autumn Biological Engineering Co., Ltd., Nanjing, Jiangsu, China) group, D-NAFLD+DIO (20 mg/kg) group and D-NAFLD+Metformin (Met, 300 mg/kg) (98% purity, Yuanye Bio-Technology Co., Ltd., Shanghai, China) group, positive control. Rats, except for the control and D-NAFLD group, were given DIO and Met by gavage daily for 8 weeks. Except for the control group, rats in the other groups were given HFD ad libitum during the intervention period. The animal experimental process is shown in Figure 1B. All animal procedures were performed following the Guide for the Care and Use of Laboratory Animals: Eighth Edition (ISBN-10:0-309-15396-4) and approved by the Animal Ethics Committee of Northwest A&F University, and Chengdu Dossy Experimental Animals Co., Ltd. (N20071065).

### 2.2. Biochemical Measurement

At the end of the experiment, the rats were anesthetized by intraperitoneal injection of 3% pentobarbital sodium (30 mg/kg). The blood was drawn from the heart and then centrifugated to collect serum (3000 × *g*, 15 min). The levels of TG, total cholesterol (TC), FFA, low-density lipoprotein (LDL), high-density lipoprotein (HDL), alanine aminotransferase (ALT), and aspartate aminotransferase (AST) were measured by corresponding assay kits purchased from Jiancheng Bioengineering Institute (Nanjing, Jiangsu, China).

### 2.3. Measurement of Insulin Resistance Index

The method for serum preparation is the same as in 2.2. An ELISA kit (Jianglai Biological Technology, Shanghai, China) was used to measure the fasting insulin level. Briefly, 10 uL serum was added to the detection holes containing 40 uL sample diluent and then incubated at 37 °C for 30 min. Then the holes were washed with scrubbing solution for 30 s and then patted dry, repeated 5 times. Subsequently, 50 uL enzyme labeled reagent was added and incubated at 37 °C for 30 min. After washing, the chromogenic solution was added and incubated at 37 °C for 15 min in the dark. After reaction termination, the absorbance at 450 nm was measured using a multifunctional microplate reader (Inffnite M200Pro, Männedorf, Switzerland). The insulin resistance index was calculated with the formula: Fasting blood glucose × fasting insulin/22.5.

### 2.4. Hematoxylin and Eosin (H&E) and Immunohistochemistry Assay

After euthanasia, the liver and pancreas were processed, including 10% neutral formaldehyde fixing, paraffin embedding, 5 µm thickness sectioning, and H&E staining [26]. The pathological changes were observed using a stereomicroscope (SMZ25, Nikon, Tokyo, Japan). For immunohistochemistry, the sections of the liver were antigen retrieval, blocked with goat serum (37 °C for 20 min), and then incubated with primary antibodies. The antibodies included AMPK (1:100, Beyotime, Shanghai, China), Phosphorylated protein kinase RNA-like endoplasmic reticulum kinase (p-PERK, 1:200, Bioss, Beijing, China), Dynamic-related protein 1 (DRP1, 1:100, Beyotime), Cytochrome c (CytC, 1:100, Proteintech, Chicago, IL, USA), caspase 12 (1:100, Bioss), Inositol-requiring enzyme-1 (IRE1, 1:100, Proteintech) and Mitofusion 1/2 (MFN1/2, 1:100, Proteintech). Subsequently, the slides were visualized using DAB and then observed under a stereomicroscope (SMZ25, Nikon).

### 2.5. Real-Time PCR Analysis

The total RNA of liver tissue was extracted using Ultrapure RNA Kit (CWBIO, Beijing, China). The RNA was reverse-transcribed into cDNA using HiFiScript cDNA Synthesis Kit (with gDNA Removal, CWBIO). The mRNA expression was analyzed by the semiquantitative real-time PCR system (Bio-Rad, Hercules, CA, USA) with SYBR reagent (CWBIO). The primers are shown in Table 1. The relative gene expression was calculated by the 2^−ΔΔCt^ method.

### 2.6. ROS Measurement

ROS was measured by DHE probe (Sigma, St. Louis, MO, USA). In detail, the liver was quickly taken out, embedded in an optimal cutting temperature compound, rapidly frozen in liquid nitrogen, and then sectioned at 5 µm. The sections were incubated with the DHE probe (37 °C for 30 min), washed with PBS, and photographed under an inverted fluorescence microscope (Lecia DMI8, Weztlar, Germany).

### 2.7. Measurement of Antioxidant Enzyme Activities

Liver antioxidant enzyme activities, including Superoxide dismutase (SOD), Catalase (CAT), and Glutathione peroxidase (GPx) were measured by corresponding commercial kits from Beyotime. Protein level was measured using bicinchoninic acid (BCA) protein assay kit (CWBIO). The activities of SOD, CAT, and GPx are presented as units/mg protein.

### 2.8. Measurement of Lipid Peroxidation

The detection of lipid peroxidation was performed by Lipid Peroxidation MDA Assay Kit (Beyotime). Protein content was also detected. The MDA level is presented as nmol/mg pro.

### 2.9. Western Blot Assay

The protein extraction and western blot were performed according to the methods previously described [24]. The primary antibodies include SREBP1 (1:1000, Beyotime), ACC (1:1000, Proteintech), p-ACC (Ser^79^, 1:1000, Proteintech), AMPK (1:1000, Beyotime), p-AMPK (Ser^172^, 1:1000, CST), Protein kinase RNA-like endoplasmic reticulum kinase (PERK, 1:1000, Bioss), p-PERK (Thr^980^, 1:1000, Bioss), Phosphorylated eukaryotic initiation factor 2 (p-EIF2α at Ser^51^, 1:1000, Beyotime), IRE1 (1:1000, Proteintech), p-IRE1 (Ser^724^, 1:1000, Bioss), X-box binding protein 1 spliced (XBP1s, 1:1000, Beyotime), Activating transcription factor 4 (ATF4, 1:1000, Proteintech), C/EBP homologous protein (CHOP, 1:1000, Proteintech), p-CHOP (Ser^30^, 1:1000, Bioss), B-cell lymphoma-2 (Bcl2, 1:1000, Proteintech), Bcl2-associated X (Bax, 1:1000, Proteintech), CytC (1:1000, Proteintech), Apoptotic protease activating factor-1 (Apaf-1, 1:1000, Proteintech), caspase 9 (1:1000, Proteintech), caspase 3 (1:1000, Proteintech), DRP1 (1:1000, Beyotime), p-DRP1 (Ser^616^, 1:1000, SAB), MFN1 (1:1000, Proteintech), MFN2 (1:1000, Proteintech), Fission 1 (FIS1, 1:1000, Proteintech), ACTB (1:1000, Beyotime) and GAPDH (1:1000, Proteintech).

### 2.10. Statistical Analysis

All data were expressed as the mean ± SD. Significant differences between different groups were determined by one-way factorial analysis of variance (ANOVA), followed by Duncan’s for multiple-range test using SPSS 20.0. * *p* < 0.05 indicates significant difference and ** *p* < 0.01 indicates a highly significant difference.

## 3. Results

### 3.1. DIO Reduced Insulin Resistance and Improved Dyslipidemia in D-NAFLD Rats

Insulin resistance and elevated blood lipid are two main pathogenic factors of D-NAFLD. Thus, insulin resistance index and blood lipid were measured. As shown in Figure 1C, the increase in insulin resistance index was inhibited by DIO and Met treatment. Intragastric administration with DIO and Met to D-NAFLD rats decreased TG, TC, FFA, and LDL levels, while the content of HDL increased after DIO and Met treatment (Figure 1D–H). These results indicated that DIO ameliorated insulin resistance index and blood lipid homeostasis in D-NAFLD rats.

### 3.2. DIO Relieved Pancreatic Injury and Mitochondrial Apoptosis in D-NAFLD Rats

**Figure 2:**
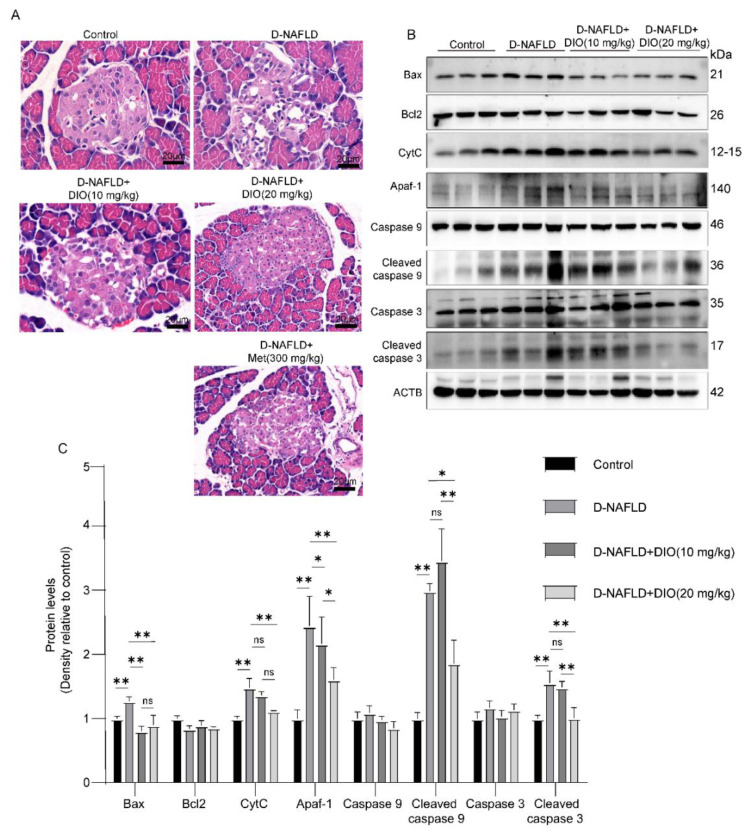
DIO relieved pancreatic injury and mitochondrial apoptosis in D-NAFLD rats. (**A**) H&E staining of pancreas, ×200. (**B**) Western blot images of Bax, Bcl2, CytC, Apaf-1, caspase 9, and caspase 3 in pancreas. (**C**) Relative protein expressions of Bax, Bcl2, CytC, Apaf-1, caspase 9, cleaved caspase 9, caspase 3, and cleaved caspase 3 in pancreas. *n* = 3 and the data were presented as mean ± SD. ns indicates no significance, * and ** indicate significant difference and highly significant difference, respectively.

Prolonged insulin resistance will impair islet function, mainly through damaging islet β cells. In D-NAFLD rats, the Langerhans cells are irregular and partially absent. DIO treatment improved the structure of islets (Figure 2A). β cell failure and subsequent apoptosis are common features of T_2_DM. As shown in Figure 2B,C, mitochondrial apoptosis-related proteins including Bax, CytC, Apaf-1, cleaved caspase 9, and cleaved caspase 3 were upregulated in the pancreas of D-NAFLD rats. After DIO treatment, their expressions decreased significantly. But no significant change in Bcl2 expression was recorded among the control, D-NAFLD, and DIO treatment groups. In sum, DIO relieved pancreatic injury and apoptosis in D-NAFLD rats.

### 3.3. DIO Ameliorated Liver Injury and Lipid Deposition in D-NAFLD Rats

**Figure 3:**
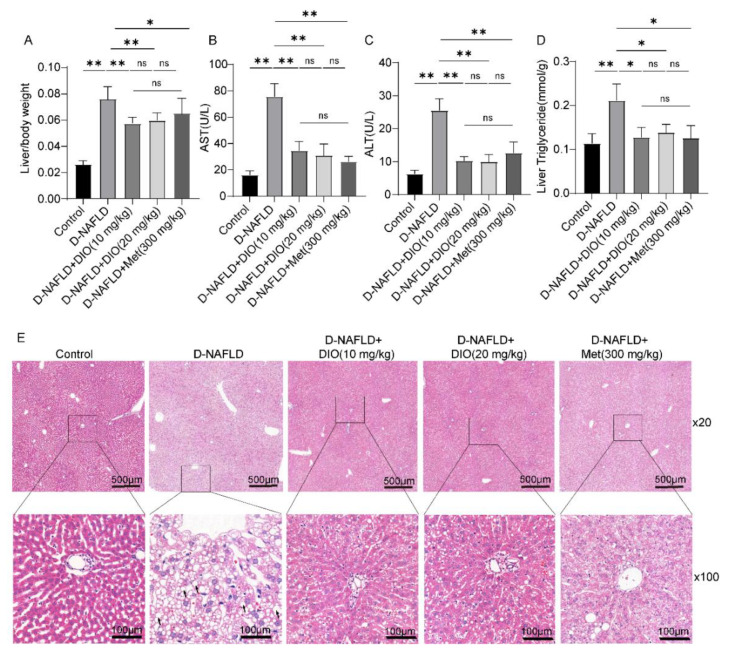
DIO ameliorated liver damage and lipid deposition in D-NAFLD rats. (**A**) Liver/body weight. (**B**) Serum AST(U/L). (**C**) Serum ALT(U/L). (**D**) Liver triglyceride(mmol/g). (**E**) H&E staining of the liver (×20 and ×100) (black arrow, lipid vacuolation). Data in (**A**–**D**) (*n* = 8) and data in (**E**) (*n* = 3) were presented as mean ± SD. * and ** indicate significant difference and highly significant difference, respectively.

Insulin resistance could increase FFA and exacerbate hepatic lipid deposition. To investigate whether DIO relieves lipid deposition and liver injury in D-NAFLD rats, liver/body ratio, serum ALT, serum AST, liver TG, and H&E staining were measured. As shown in Figure 3A, administration with DIO and Met to D-NAFLD rats decreased liver/body weight. DIO and Met also significantly alleviated the increase of AST, ALT, and liver TG levels in D-NAFLD rats (Figure 3B–D). H&E staining showed lipid vacuoles (black arrow) in the liver of D-NAFLD rats. DIO and Met decreased liver lipid accumulation (Figure 3E). The above data indicated that DIO relieved liver injury and hepatic steatosis in D-NAFLD rats.

### 3.4. DIO Inhibited DNL and Enhanced FAO via AMPK-ACC/SREBP1 and AMPK-ACC Pathways

**Figure 4:**
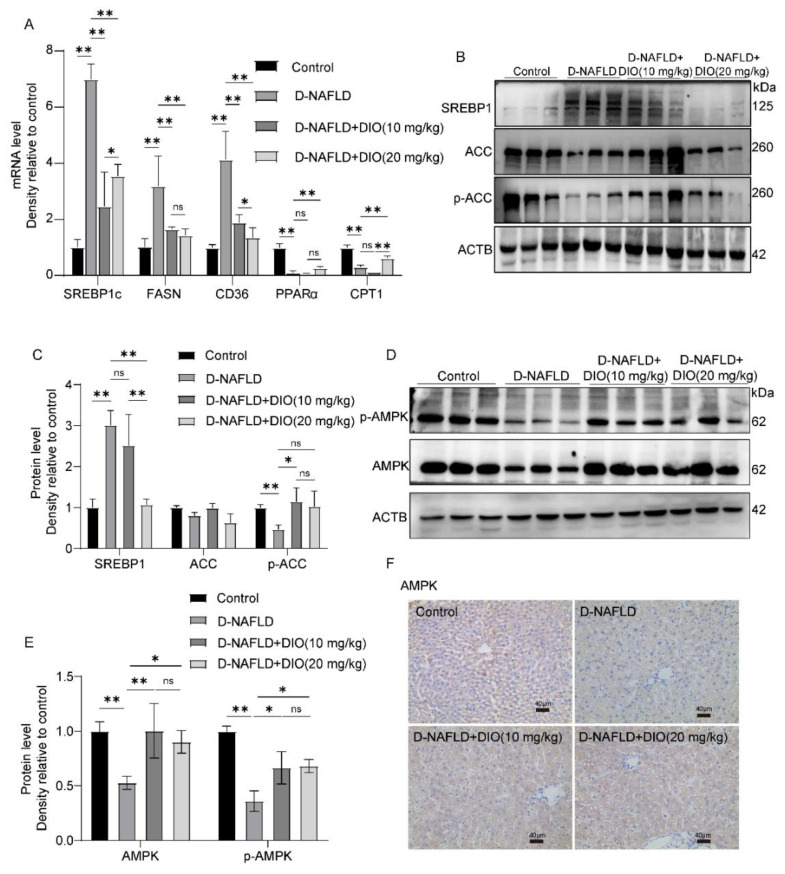
DIO inhibited hepatic steatosis through the regulation of AMPK-ACC/SREBP1 pathway. (**A**) The mRNA expressions of *SREBP1c*, *FASN*, *CD36*, *PPARα*, and *CPT1*. (**B**) Western blot images of SREBP1, ACC, p-ACC, and ACTB. (**C**) Relative protein expressions of SREBP1, ACC, and p-ACC. (**D**) Western blot images of p-AMPK, AMPK, and ACTB. (**E**) Relative protein expressions of AMPK and p-AMPK. (**F**) The images of AMPK immunohistochemistry, ×100, scale bars = 40 µm. Data in (**A**) (*n* = 5) and data in (**B**–**F**) (*n* = 3) were presented as mean ± SD. ns indicates no significance, * and ** indicate significant difference and highly significant difference, respectively.

As shown in Figure 4A, D-NAFLD rats displayed increased levels of adipogenic genes including *SREBP1c*, *FASN*, and CD36 molecule (thrombospondin receptor) (*CD36*), but decreased FAO-associated genes, including Peroxisome proliferator activated receptor α (*PPARα*) and Carnitine palmitoyltransferase-1 (*CPT1*). DIO administration decreased *SREBP1c*, *FASN*, and *CD36* expressions, and increased *PPARα* and *CPT1* expressions. ACC serves as an important precursor for the biosynthesis of fatty acid and an effective inhibitor of long-chain fatty acyl-CoA transporting into mitochondria. SREBP1 is a key transcription factor for DNL through upregulating genes coding FASN. As shown in Figure 4B,C, compared to D-NAFLD rats, DIO downregulated the expression of SREBP1 and increased p-ACC expression. AMPK plays a crucial role in regulating lipid metabolism. It can inhibit DNL and enhance FAO through the regulation of SREBP1 and ACC expressions. In D-NAFLD rats, the expressions of AMPK and p-AMPK decreased greatly, while their expressions were enhanced after DIO application (Figure 4D–F and Figure S1). The above results indicate that DIO relieved lipid deposition mainly through inhibiting DNL and enhancing FAO via AMPK-ACC/SREBP1 and AMPK-ACC pathways, respectively.

### 3.5. DIO Inhibited ER Stress through Regulating PERK and IRE1 Pathways

**Figure 5:**
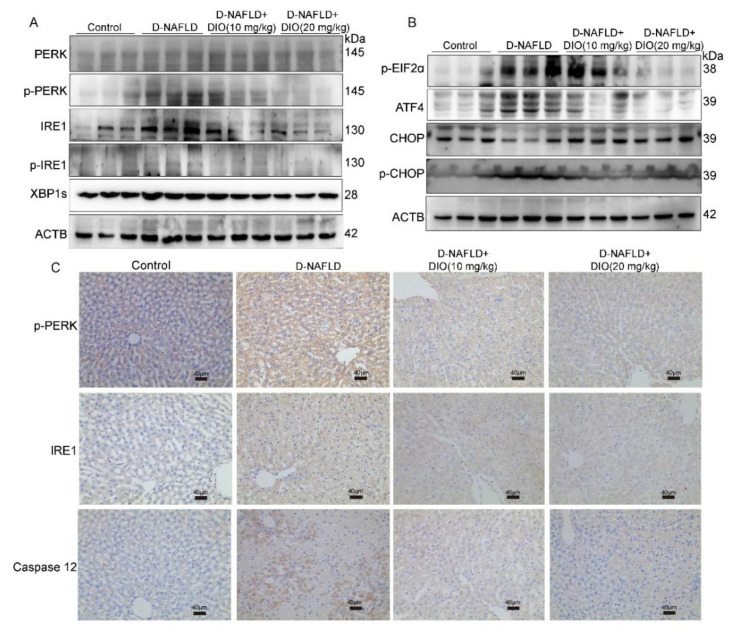
DIO ameliorated ER stress and associated apoptosis in the liver of D-NAFLD rats. (**A**) Western blot images of PERK, p-PERK, IRE1, p-IRE1, XBP1s, and ACTB. (**B**) Western blot images of p-EIF2α, ATF4, CHOP, p-CHOP, and ACTB. (**C**) The images of p-PERK, IRE1, and caspase 12 immunohistochemistry, ×100, scale bars = 40 µm.

Saturated fatty acids can alter phospholipids’ composition in the ER membrane and directly activate the sensors of IRE1 and PERK to induce ER stress, which could exacerbate hepatic steatosis through regulating DNL. Thus, the protein expressions of PERK, p-PERK, IRE1, p-IRE1, and their downstream were measured. It is shown that proteins in the PERK pathway including PERK, p-PERK, and p-EIF2α were upregulated in the liver of D-NAFLD rats (Figure 5A–C and Figure S2A,B). Similarly, the protein expressions in the IRE1 pathway including IRE1, p-IRE1, and XBP1s were increased in D-NAFLD rats (Figure 5A,C and Figure S2A,C). Their expressions decreased after the DIO administration. ATF4 and CHOP are two proteins regulating ER stress-associated apoptosis by responding to the signal from PERK and IRE1. In this study, protein expressions of p-CHOP and ATF4 increased greatly in D-NAFLD rats. DIO downregulated their expressions (Figure 5B and Figure S2D). Caspase 12 is the executor of ER stress-associated apoptosis. It was upregulated in the liver of D-NAFLD rats, but after DIO treatment, the expression of caspase 12 decreased (Figure 5C and Figure S2D,E). These data suggested that DIO protects against ER stress and its mediated apoptosis through regulating PERK and IRE1 pathways, which may inhibit DNL.

### 3.6. DIO Ameliorated Oxidative Stress and Inflammation in the Liver of D-NAFLD Rats

**Figure 6:**
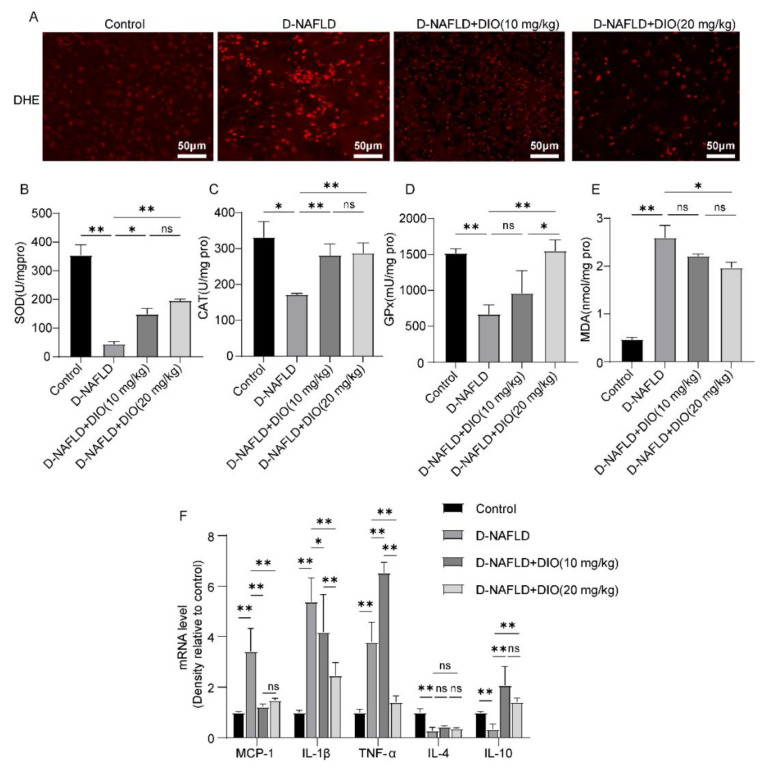
DIO ameliorated oxidative stress and inflammation in the liver of D-NAFLD rats. (**A**) DHE fluorescence, ×200, scale bars = 50 µm. (**B**) SOD level. (**C**) CAT level. (**D**) GPx level. (**E**) MDA level. (**F**) The mRNA expressions of *MCP-1*, *IL-1β*, *TNF-α*, *IL-4* and *IL-10*. Data in (**A**) (*n* = 3) and data in (**B**–**F**) (*n* = 5) were presented as mean ± SD. ns indicates no significance, * and ** indicate significant difference and highly significant difference, respectively.

Oxidative stress and inflammation can accelerate the progress of NAFLD. Thus, DHE fluorescence, the activities of antioxidant enzymes, and the expressions of inflammatory factors were measured. As shown in Figure 6A, superoxide anion increased remarkably in the liver of D-NAFLD rats, which was relieved by DIO application. The activities of SOD, CAT, and GPx decreased significantly in D-NAFLD rats; after the DIO application, their activities were enhanced. MDA is used as an indicator of lipid oxidation (Figure 6B–D). As shown in Figure 6E, the level of MDA increased remarkably, while it decreased significantly after DIO (20 mg/kg) treatment. qPCR analysis of inflammatory factors showed that the expressions of *MCP-1*, *IL-1β*, and *TNF-α* were upregulated significantly in the liver of D-NAFLD rats, while *IL-4* and *IL-10* were downregulated. DIO treatment downregulated *MCP-1*, *IL-1β* and *TNF-α* expressions, and increased *IL-10* expression, but had no significant effect on *IL-4* expression (Figure 6F). These results showed that DIO reduced ROS production, enhanced antioxidant enzyme activities, and inhibited inflammation in the liver of D-NAFLD rats.

### 3.7. DIO Ameliorated Mitochondrial Apoptosis in the Liver of D-NAFLD Rats

**Figure 7:**
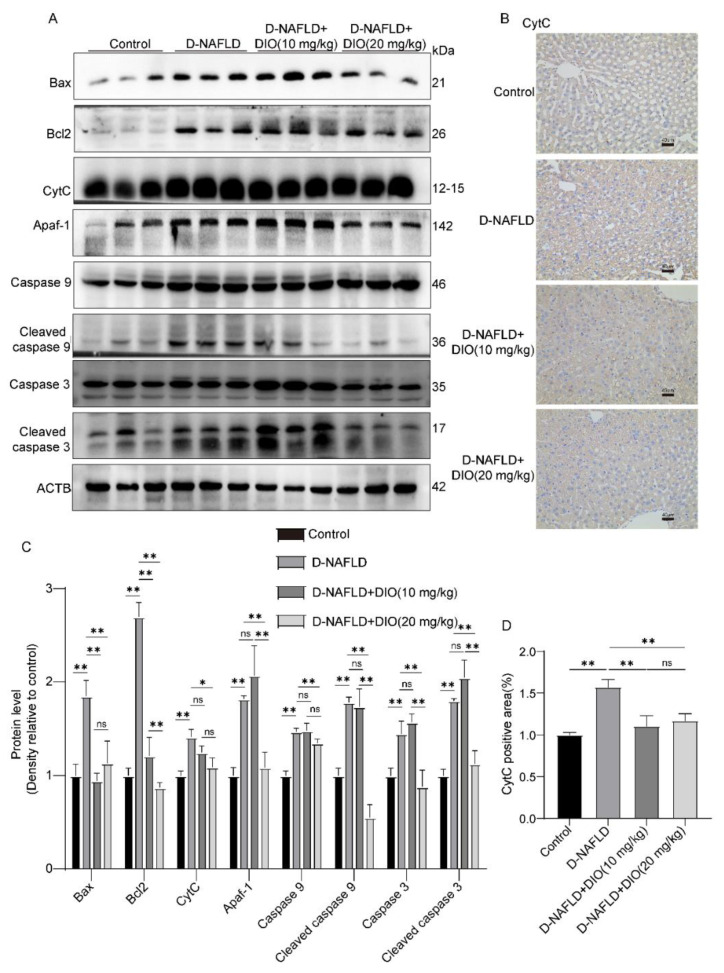
DIO ameliorated mitochondrial apoptosis in the liver of D-NAFLD rats. (**A**) Western blot images of Bax, Bcl2, CytC, Apaf-1, caspase 9, cleaved caspase 9, caspase 3, cleaved caspase 3, and ACTB. (**B**) The images of CytC immunohistochemistry, ×100, scale bars = 40 µm. (**C**) Relative protein expressions of Bax, Bcl2, CytC, Apaf-1, caspase 9, cleaved caspase 9, caspase 3, and cleaved caspase 3. (**D**) CytC positive area in immunohistochemistry. *n* = 3 and the data were presented as mean ± SD. ns indicates no significance, * and ** indicate significant difference and highly significant difference, respectively.

ROS can damage mitochondrial DNA and lead to cell apoptosis, establishing a self-perpetuating vicious cycle. It is shown that pro-apoptotic protein-Bax, apoptosis factor-CytC, and Apaf-1 were upregulated in the liver of D-NAFLD rats. After the DIO application, Bax, CytC, and Apaf-1 expressions decreased significantly (Figure 7A–D). In addition, the caspase cascade was activated in the liver of D-NAFLD rats, as1 evidenced by the increase in cleaved caspase 9 and cleaved caspase 3 expressions. DIO application to D-NAFLD rats decreased cleaved caspase 9 and cleaved caspase 3 expressions (Figure 7A,C). Collectively, these data indicated that DIO inhibited mitochondria-mediated apoptosis in the liver of D-NAFLD rats.

### 3.8. DIO Ameliorated the Disorder of Mitochondrial Fission and Fusion in the Liver of D-NAFLD Rats

**Figure 8:**
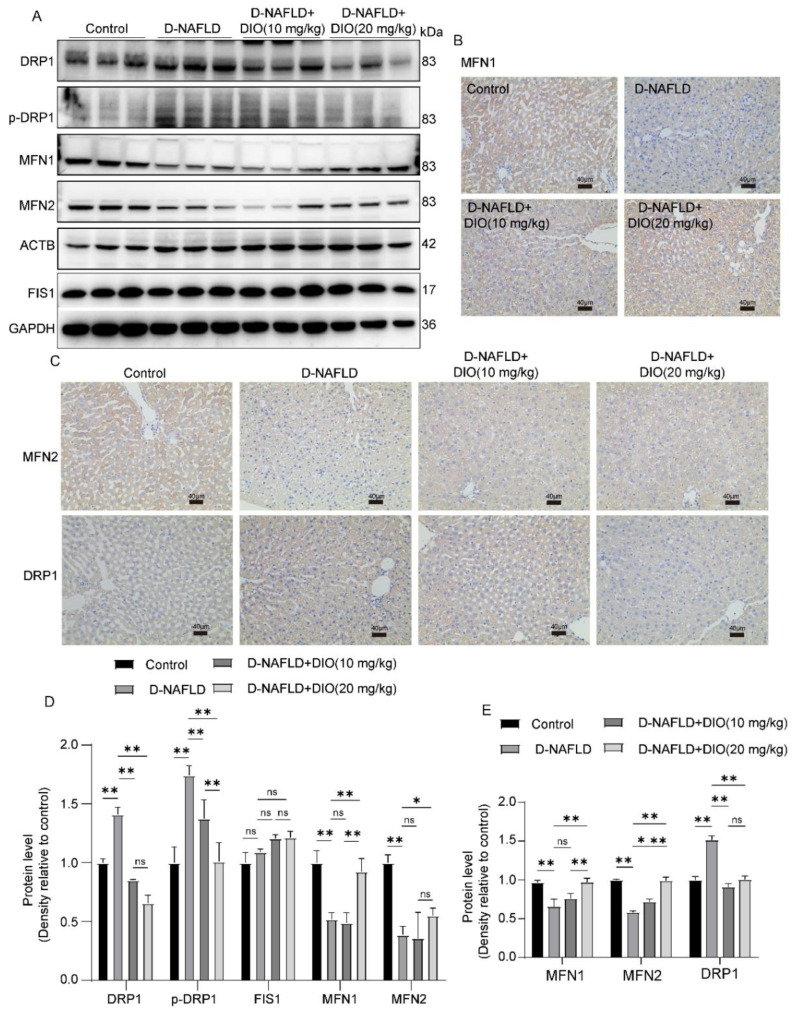
DIO ameliorated the disorder of mitochondrial fission and fusion in the liver of D-NAFLD rats. (**A**) Western blot images of DRP1, p-DRP1, MFN1, MFN2, FIS1, ACTB, and GAPDH. (**B**) The images of MFN1 immunohistochemistry, ×100, scale bars = 40 µm. (**C**) The images of MFN2 and DRP1 immunohistochemistry, ×100, scale bars = 40 µm. (**D**) Relative protein expressions of DRP1, p-DRP1, FIS1, MFN1, and MFN2. (**E**) Positive staining area of MFN1, MFN2, and DRP1 immunohistochemistry. *n* = 3 and the data were presented as mean ± SD. ns indicates no significance, * and ** indicate significant difference and highly significant difference, respectively.

Decreasing mitochondrial fission represents a promising therapeutic target for NAFLD. In this study, the expressions of DRP1 and p-DRP1, proteins regulating mitochondrial fission, were greatly upregulated in the liver of D-NAFLD rats (Figure 8A,C–E). No significant change was recorded in FIS1 expression in the rats of D-NAFLD and DIO treatment groups. While mitochondrial fusion proteins including MFN1 and MFN2 were downregulated significantly in the liver of D-NAFLD rats (Figure 8). DIO application decreased DRP1 and p-DRP1 expressions but increased MFN1 and MFN2 expressions (Figure 8). The above data suggested that DIO inhibited mitochondrial fission and enhanced mitochondrial fusion in the liver of D-NAFLD rats.

## 4. Discussion

T_2_DM is associated with various liver abnormalities, especially NAFLD. The risk of NAFLD in T_2_DM patients is two to three times higher than that in healthy people. The mechanism of D-NAFLD is complex, including insulin resistance, the disorder between hepatic lipid uptake, DNL and FAO, ER stress, oxidative stress, mitochondrial dysfunction, etc. [4,9,27]. The potent role of DIO in the prevention of NAFLD has been reported previously. Khateeb et al. [28] indicated that DIO downregulated the expressions of fatty acid synthesis genes: SREBP1 and FASN in the liver of HFD-induced obesity mice. Fang et al. [29] suggested that DIO relieved lipid accumulation through inhibiting oxidative stress and lipid synthesis via the inhibition of SREBP-1c/FASN pathway in LO2 cells treated with palmitic acid. However, the concrete mechanism by which DIO improves hepatic steatosis under T_2_DM is still unclear.

Elevated blood lipid and insulin resistance are two main pathogenic factors of D-NAFLD. In this study, dyslipidemia was ameliorated by DIO and Met through decreasing TG, TC, FFA, and LDL levels, and increasing HDL content. In addition, DIO and Met decreased the insulin resistance index and improved pancreatic structure. Lipotoxicity and glucotoxicity of T_2_DM can cause pancreatic β-cell apoptosis and reduced pancreatic mass and function. In this study, DIO inhibited pancreatic damage through inhibiting mitochondrial-mediated apoptosis, especially DIO (20 mg/kg). It is documented that insulin resistance could lead to hepatic steatosis and the elevated serum ALT and AST are indicators of liver damage. In the present study, the levels of serum ALT and AST increased significantly in D-NAFLD rats; after DIO and Met treatment, they decreased greatly. The results of H&E staining and liver triglyceride consistently showed that DIO and Met decreased hepatic steatosis. In addition, administration with DIO and Met to D-NAFLD rats reduced liver/body weight. The effects of DIO and Met on the above results were equivalent and there was no statistical significance between DIO (10 mg/kg) and DIO (20 mg/kg) treatments, except for pancreatic cell apoptosis (20 mg/kg DIO is better).

In this study, Met is used as a positive control. Since our aim is to explore the protective effect of DIO on D-NAFLD, mechanistically, we only focused on DIO. One of the pathogeneses of hepatic steatosis is the disorder between hepatic lipid uptake, DNL, and FAO. Emerging evidence has indicated that AMPK plays a crucial role in NAFLD by stimulating FAO and inhibiting lipogenesis. Once activated, AMPK induces the phosphorylation of ACC at Ser^79^ and then its inactivation. ACC serves as an important precursor for the biosynthesis of fatty acid and an effective inhibitor of long-chain fatty acyl-CoA transporting into mitochondria [30,31]. In addition, AMPK can phosphorylate SREBP1 at Ser^372^, suppressing the proteolytic cleavage of precursor SREBP1 into mature SREBP1, which then reduces FASN expression and inhibits lipid synthesis [13]. We showed that the DIO application enhanced the protein expressions of AMPK and p-AMPK. The phosphorylation of ACC1 at Ser^79^ was also upregulated greatly after DIO administration. Moreover, DIO inhibited the over-expression of SREBP1 in D-NAFLD rats. In addition, the mRNA expressions of lipogenic genes including *SREBP1*, *FASN*, and *CD36* decreased greatly. The suppression of genes involving in β-oxidation such as *PPARα* and *CPT1* impairs the metabolic function of mitochondria [32,33]. In this study, the mRNA expressions of *PPARα* and *CPT1* decreased greatly in the liver of D-NAFLD rats, which was enhanced by DIO. These results indicated that DIO can reduce lipid synthesis and enhance FAO. In general, there was no significant difference between the two concentrations of DIO.

ER stress is also a therapeutic target for NAFLD. It can be directly activated by glucolipotoxicity. Saturated fatty acids alter phospholipids’ composition in the ER membrane and then trigger ER stress through the sensors of IRE1α and PERK even in the absence of unfolded proteins [34,35]. Studies have indicated that both IRE1α-XBP1 and PERK-EIF2 arms participate in hepatic steatosis through the regulation of DNL, lipogenesis, and very low-density lipoprotein secretion [14,36,37]. The IRE1α-XBP1 pathway can trigger de novo lipogenesis (DNL) by SREBP1 and its downstream genes [38]. Cho et al. [39] suggested that allopurinol relieved hepatic steatosis induced by high-fructose diet through the modulation of ER stress via the IRE1 pathway. Wu et al. [40] showed that Patchouli alcohol attenuated ER stress and hepatic steatosis through inhibiting the activation of PERK and IRE1 pathways in HFD-fed rats. Our study showed that the expressions of proteins involved in PERK and IRE1 arms were upregulated greatly in the liver of D-NAFLD rats. DIO administration decreased their expressions. In addition, proteins associated with apoptosis mediated by ER stress, including ATF4, p-CHOP, and caspase 12 were also downregulated by DIO. The inhibitory effects of DIO (20 mg/kg) on ER stress were better than that of DIO (10 mg/kg).

Mitochondria is the main site for FAO in cells. Per hepatocyte is thought to contain 500 to 4000 mitochondria, and a decrease in their capacity to oxidize fatty acids contributes to the development of hepatic steatosis [41]. As an adaptive process, the accumulation of FFAs enhanced mitochondrial FAO, the tricarboxylic acid cycle, and oxidative phosphorylation, and meanwhile, ROS was produced. The persistent ROS production attacks polyunsaturated fatty acids, leading to the production of aldehyde by-products including MDA, which amplifies oxidative stress. In this condition, the dysfunction of FAO, mtDNA damage, and mitochondria-mediated apoptosis occur. The apoptosis begins with the collapse of mitochondrial membrane potential and the release of apoptotic factors such as CytC and AIF, followed by the formation of apoptosis complex and caspase cascade activation. In the present study, the elevation of ROS and MDA levels was observed in the liver of D-NAFLD rats, which was ameliorated by DIO treatment. In addition, administration with DIO to D-NAFLD rats enhanced antioxidant enzyme activities and inhibited inflammation through regulation of the expressions of inflammatory factors. Moreover, mitochondria-mediated apoptosis was suppressed by decreasing Bax, CytC, and Apaf-1 expressions and preventing the activation of the caspase cascade. The mitigative effects of DIO (20 mg/kg) on oxidative stress and mitochondrial apoptosis were better than that of DIO (10 mg/kg).

Mitochondria continuously undergo fusion and fission. Various studies have suggested that excessive mitochondrial fission is involved in hepatic steatosis and liver inflammation, which results in hepatocyte death [30,42,43,44]. For example, the expressions of MFN1, MFN2, and OPA1 decreased greatly in the liver of SD rats fed with HFD for 12 weeks and their expressions increased following RYGB surgery [45]. Hernández et al. [46] indicated that a high sucrose diet increased liver mitochondrial fission by upregulating DRP1 expression in Wistar rats. In the present study, DIO reduced DRP1 and p-DRP1(Ser^616^) expressions. To the contrary, MFN1 and MFN2 expressions were upregulated greatly after the DIO application. It is worth noting that the changes of p-DRP1, MFN1, and MFN2 were more significant in the D-NAFLD+DIO (20 mg/kg) group.

## 5. Conclusions

In conclusion, the current study revealed that DIO can protect against NAFLD in T_2_DM rats induced by HFD and STZ injection and that DIO (20 mg/kg) is the preferred option. Firstly, DIO reduced the insulin resistance index and relieved pancreatic damage and apoptosis. Then, DIO improved dyslipidemia and hepatic steatosis, mainly through suppressing DNL and enhancing FAO via AMPK-ACC/SREBP1 and AMPK-ACC pathways. DIO also relieved ER stress, which may then inhibit DNL and improve mitochondrial function, a site for FAO. Thus, the dietary intervention of DIO has great potential in relieving D-NAFLD, which can help or even substitute for medication, and it is valuable to study the synergistic effect of DIO with drugs such as Met.

## Figures and Tables

**Table 1:** The primer sequences for the targeted genes.

Primers	Forward Sequence (5′-3′)	Reverse Sequence (5′-3′)
*SREBP1*	ACCCTGCGAAGTGCTCACAA	GCGTTTCTACCACTTCAGGTTTCA
*FASN*	GAGCGTTCGTGAAACCGACA	AGGTTGGTGCACCTCCACTTG
*CD36*	CGGCGATGAGAAAGCAGA	ACTCCAACACCAAGTAAGACCA
*CPT1*	CTGCTGTATCGTCGCACATTAG	GTTGGATGGTGTCTGTCTCTTCC
*PPARα*	GCTCTGAACATTGGCGTTCG	TCAGTCTTGGCTCGCCTCTA
*IL-1β*	CCTTGTGCAAGTGTCTGAAGC	CCCAAGTCAAGGGCTTGGAA
*IL-4*	AACACCACGGAGAACGAGCTCATC	AGTGAGTTCAGACCGCTGACACCT
*TNF-α*	CTCCAGCTGGAAGACTCCTCCCAG	CCCGACTACGTGCTCCTCACC
*IL-10*	AGAAGAGGGAGGAGCCTTTG	GCCTTTGCTGGTCTTCACTC
*MCP-1*	TGCTGTCTCAGCCAGATGCAGTTA	AGAAGTGCTTGAGGTGGTTGTGGA
*Actb*	TGACAGGATGCAGAAGGAGA	TAGAGCCACCAATCCACACA

## Data Availability

The data presented in this study are available on request from the corresponding author.

## Supplementary Materials

The following supporting information can be downloaded at: https://www.mdpi.com/article/10.3390/nu14234994/s1, Figure S1: DIO increased AMPK expression in the liver of D-NAFLD rats; Figure S2: DIO ameliorated ER stress and associated apoptosis in the liver of D-NAFLD rats.
