# Supplementary material for: Diosgenin Ameliorated Type II Diabetes-Associated Nonalcoholic Fatty Liver Disease through Inhibiting De Novo Lipogenesis and Improving Fatty Acid Oxidation and Mitochondrial Function in Rats
Source: Nutrients. 2022 Nov 24;14(23):4994. doi: 10.3390/nu14234994 (PMC9738614; doi:10.3390/nu14234994)
Supplement: Supplementary file 1 [file nutrients-14-04994-s001.zip › nutrients-2013337-supplementary.pdf]

Supplementary Materials

Diosgenin Ameliorated Type II Diabetes-Associated Nonalcoholic Fatty Liver Disease through Inhibiting De Novo Lipogenesis and Improving Fatty Acid Oxidation and Mitochondrial Function in Rats

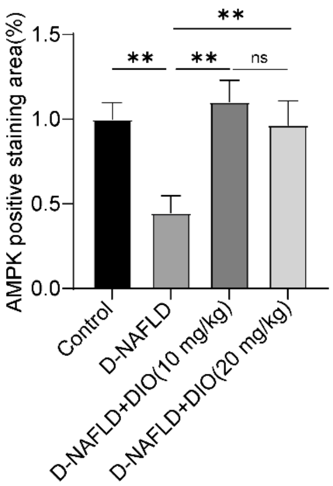

Figure S1. DIO increased AMPK expression in the liver of D-NAFLD rats. AMPK positive area in immunohistochemistry. n = 3 and the data were presented as mean ± SD. * and ** indicate significant difference and highly significant difference, respectively.

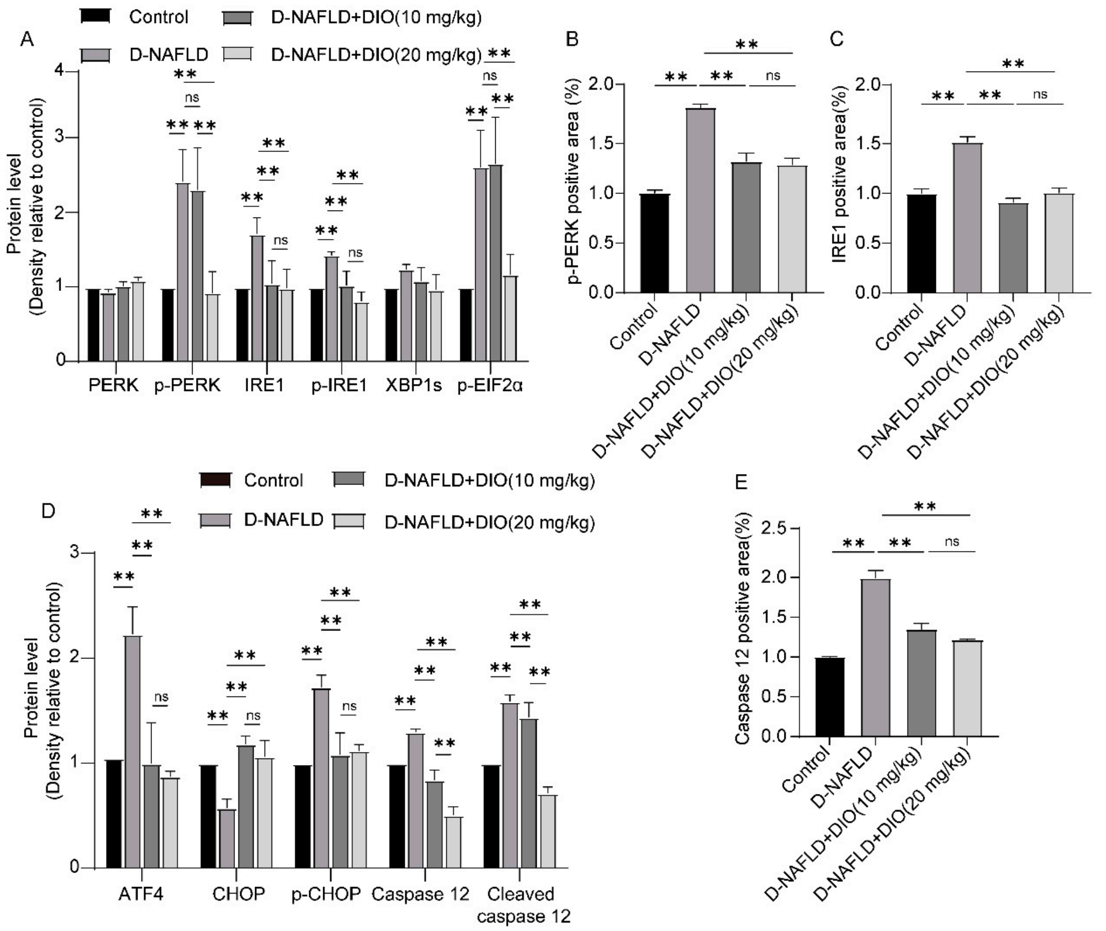

Figure S2. DIO ameliorated ER stress and associated apoptosis in the liver of D-NAFLD rats. (A) Relative protein expressions of PERK, p-PERK, IRE1, p-IRE1, XBP1s, and p-EIF2α. (B) p-PERK positive area in immunohistochemistry. (C) IRE1 positive area in immunohistochemistry. (D) Relative protein expressions of ATF4, CHOP, p-CHOP, caspase 12, and cleaved caspase 12. (E) Caspase 12 positive area in immunohistochemistry. $n = 3$ and the data were presented as mean ± SD. ns indicates no significance, * and ** indicate significant difference and highly significant difference, respectively.
